# HIV prevalence among transmasculine individuals at a New York City Community Health Centre: a cross‐sectional study

**DOI:** 10.1002/jia2.25981

**Published:** 2022-10-12

**Authors:** Asa E. Radix, Elaine L. Larson, Alexander B. Harris, Mary Ann Chiasson

**Affiliations:** ^1^ Department of Medicine Callen‐Lorde Community Health Center New York City New York USA; ^2^ Department of Epidemiology Columbia University Mailman School of Public Health New York City New York USA; ^3^ Division of Infectious Diseases, Department of Medicine Columbia University Irving Medical Center New York City New York USA

**Keywords:** transgender, HIV, transgender men, transmasculine, HIV risk, gender diverse

## Abstract

**Introduction:**

Multiple studies have demonstrated elevated incidence and prevalence of HIV among transgender women; however, few studies have been conducted among transmasculine individuals. HIV prevalence among transgender men in the United States is estimated to be 0–4%; however, there have not been any US studies examining HIV prevalence that stratify by the gender of sexual partners. The aim of this research was to examine HIV prevalence and its association with socio‐demographic and other factors, including the gender of sexual partners and receipt of gender‐affirming care (hormones/surgery), among transmasculine individuals at the Callen‐Lorde Community Health Center in New York City.

**Methods:**

The Transgender Data Project was an Institutional Review Board‐approved retrospective chart review of all transgender and gender diverse clients at the clinic, ages 18+, between 1 January 2009 and 12 December 2010. Charts were reviewed manually. Data included birth sex, gender, race/ethnicity, education, employment, housing, insurance status, gender of sexual partners, HIV screening and status, and receipt of gender‐affirming care. Bivariate and multivariable logistic regression models were used to assess the association between HIV status and other variables.

**Results and discussion:**

Five hundred and seventy‐seven transmasculine individuals, mean age 32.1 years (18.3–70.5), were included in this analysis. A small majority were White (55% White, 13.9% Black and 11.7% Hispanic). The majority, 78.9%, had received hormones (testosterone) and 41.6% had received at least one gender‐affirming surgery. The HIV screening rate was 43.4%. HIV prevalence was 2.8%, (95% CI: 1.13%, 5.68%) among those screened, notably higher than the US population prevalence. HIV prevalence was highest among transmasculine individuals who had sex exclusively with cisgender men (11.1%). In the multivariable model (age, education and gender of sexual partners), the adjusted odds ratio of HIV for those who had sex exclusively with cisgender male partners compared to no cisgender male partners was 10.58 (95% CI: 1.33, 84.17).

**Conclusions:**

Although HIV prevalence has been estimated to be low among transgender men, the analysis found heterogeneous results when stratified by gender of sexual partners. The results underscore the need to understand sexual risk among transmasculine individuals and to disaggregate HIV data for those having sex with cisgender men, thus also allowing for better inclusion in HIV prevention efforts.

## 1 INTRODUCTION

There are an estimated 1 million transgender adults in the United States [1] who are heterogeneous in terms of age, race, ethnicity, socio‐economic status, gender and sexual orientation identities [2, 3]. Over the last decade, there has been a heightened awareness of the many health disparities faced by transgender people, including the high prevalence of HIV, sexually transmitted infections (STIs), substance use and mental health conditions that are mainly driven by a complex array of individual, interpersonal and structural factors [4, 5, 6]. Despite rapid growth in transgender health research, especially HIV‐related research, much of this has been conducted in populations of transgender women with very few studies examining HIV prevalence, risk and prevention among transgender men [5, 7, 8]. Multiple studies have demonstrated elevated HIV incidence and prevalence among transgender women, including an often‐cited estimated global prevalence of 19% [9, 10, 11, 12]. In the United States, a systematic review estimated a prevalence of 14% among transgender women, with rates two‐ to three‐fold higher among Hispanic and Black transgender women [13]. HIV prevalence among transgender men in the same study was estimated to be 3.2% (95% CI: 1.4%, 7.1%); however, insufficient data did not allow stratification by risk factor, that is transgender men who have sex with men (TMSM) versus transgender men who have sex with cisgender women, or race/ethnicity [13]. Despite these limited data, it is evident that TMSM, similar to cisgender MSM, may engage in sexual behaviours that are associated with increased risk of HIV acquisition, such as condomless anal receptive sex, sex with partners who are HIV positive or of unknown status and substance use during sex (chem sex), placing them at heightened risk for HIV and STI acquisition [13, 14, 15, 16, 17, 18]. Understanding factors related to HIV acquisition among transgender men is important and can lay the foundation for appropriate and targeted prevention interventions. This remains an important gap in the literature. This study describes the prevalence of HIV among transmasculine clients at the Callen‐Lorde Community Health Center, a healthcare clinic in New York City that predominately serves the lesbian, gay, bisexual, transgender and queer communities. The objective of this study was to investigate HIV prevalence among transmasculine individuals and its association with socio‐demographic factors and receipt of gender‐affirming medical interventions.

## 2 METHODS

This study was an observational retrospective chart review. Data were extracted from the electronic health record (EHR) at Callen‐Lorde. The inclusion criteria were (1) registered clients with medical visits between 1 January 2009 and 12 December 2010, (2) transgender identity and (3) age ≥18 years. The analysis in this manuscript only includes transgender individuals assigned female at birth. The Clinical Directors Network, Inc's Institutional Review Board approved study activities and granted a waiver of informed consent (004‐11E).

### 2.1 Measures

Gender identity was ascertained with an algorithm using (1) ICD‐9 codes 302.85 (gender identity disorder) or 259.9 (unspecified endocrine disorder), (2) mismatch between legal gender and assigned birth sex, (3) self‐reported transgender status, (4) discordant legal name and preferred name, and (5) designated female receiving testosterone treatment. All charts with at least one of these underwent chart review by two reviewers to verify gender identity and birth sex. Race, ethnicity, income, insurance status, education and housing status were obtained from designated demographic fields in the EHR. Receipts of gender‐affirming hormone therapy and surgery (GAHT and GAS) were obtained from the medical and surgical history and prescription records. The genders of sexual partners were identified from a sexual health template that was used for those undergoing HIV screening. This included data on substance use and whether sexual partners were cisgender men, cisgender women, transgender men or transgender women. There was no option for nonbinary sexual partners in the EHR template, as this identity and term were less recognized at the time. Additionally, there were no recorded data on the number of sexual partners, or sexual behaviours (whether engaging in vaginal/anal/oral sex). Receipt of HIV/STI screening and results were obtained from HIV testing fields and laboratory orders and results. The variables were dichotomized for the analysis as follows: gender‐affirming hormones—receipt of GAHT ever (yes/no); gender‐affirming surgeries—receipt of any GAS (including phalloplasty, metoidioplasty, chest reconstruction or top surgery and hysterectomy/oophorectomy) (yes/no); education—less than high school diploma or ≥ high school diploma; substance use—a record of a diagnosis of substance use ever in EHR (yes/no); and employment—current employment status documented in the medical record (employed/unemployed). Transmasculine individuals who have sex with cisgender men belong to a heterogeneous group, including those who predominately partner with cisgender women, and others who predominately partner with cisgender men. To minimize uncertainty in the assessment of risk, the variable “sex with cisgender men only” (yes/no) was created that compared those with cisgender male partners only to those without a history of cisgender male partners.

### 2.2 Statistical analysis

Chi‐square tests were used to examine differences in expected and observed proportions by HIV screening status. Crude odds ratios (ORs) were estimated for associations between HIV status and socio‐demographic, behavioural and health variables among those who had been screened for HIV (*n* = 250). We used multivariable logistic regression to estimate the adjusted odds of an HIV diagnosis by age and the variables that were significant at *p*<0.1 in the bivariate analyses. This model included 220 complete cases. The Hosmer–Lemeshow goodness of fit test was performed for model fitness (*p*>0.05). All *p*‐values are two‐tailed at a significance level of 5%. Analyses were performed using IBM SPSS Statistics for Windows, Version 26.0.

## 3 RESULTS AND DISCUSSION

A total of 3197 records were retrieved and reviewed, of these 1670 of records were verified as being transgender clients. This analysis is restricted to the 577 transgender individuals assigned female at birth, whose identities included transgender men, FTM (female‐to‐male), transsexual men, gender non‐conforming and genderqueer. In view of these diverse identities, the term “transmasculine” will be used in this analysis.

### 3.1 Patient characteristics

Table 1 presents frequencies of socio‐demographic characteristics and utilization of gender‐affirming care for the 577 transmasculine individuals in the sample. The mean age was 32.15 years, (SD 9.31, range 18.3–70.5). A small majority were White (55.0%); 95.1% had attained at least a high school diploma, 33.1% 4‐year college degree and 18.7% held a graduate degree. Over one quarter was unemployed (28.7%) and 12.2% were uninsured. Most were stably housed (96.6%) versus unstable/homeless. The low rates of housing instability and high proportion with health insurance in this group differed from Callen‐Lorde's usual patient demographics. In 2012, approximately 47% of Callen‐Lorde's patient population was uninsured. Due to a dearth of gender‐affirming care available in the city, many transgender and gender diverse individuals seek care in this “safety net” health centre, and demographic factors, such as housing and insurance, may not be typical of the traditional populations served by US community health centres.

**Table 1:** Demographic and other socio‐economic variables among transmasculine clients

Variable	All patients *N* = 577 *N* (%) or mean (SD)	No HIV screen *N* = 327 *N* (%) or mean (SD)	HIV screened *N* = 250 *N* (%) or mean (SD)	*p*‐value
Mean age in years (SD)	32.2 (9.31)	31.4 (9.76)	33.2 (8.58)	0.017
Range	(18.3–70.5)	(18.3–70.5)	(18.8–58.9)	
Race/Ethnicity	*n* = 496	*n* = 274	*n* = 222	0.000
Hispanic	58 (11.7)	27 (9.9)	31 (14.0)	
White	273 (55.0)	173 (63.1)	100 (45)	
Black	69 (13.9)	25 (9.1)	44 (19.8)	
Asian/Pacific Islander	29 (5.8)	17 (6.2)	12 (5.4)	
Other/multiracial	67 (13.5)	32 (11.7)	35 (15.8)	
Education (highest level)	*n* = 493	*n* = 273	*n* = 220	0.831
Less than high school	24 (4.9)	12 (4.4)	12 (5.5)	
High school diploma	58 (11.8)	30 (11.0)	28 (12.7)	
Some college	156 (31.6)	86 (31.5)	70 (31.8)	
Bachelors’ degree	163 (33.1)	97 (35.5)	66 (30.0)	
Graduate degree	92 (18.7)	48 (17.6)	44 (20.0)	
History of substance use	18 (3.9)	3 (0.9)	15 (6.0)	0.001
Employment	(*n* = 540)	(*n* = 302)	(*n* = 238)	0.251
Employed	385 (71.3)	209 (69.2)	176 (73.9)	
Unemployed	155 (28.7	93 (30.8)	62 (26.1)	
Housing	(*n* = 526)	(*n* = 292)	(*n* = 234)	0.639
Stable	508 (96.6)	283 (96.9)	225 (96.2)	
Unstable	18 (3.4)	9 (3.1)	9 (3.8)	
Insurance	(*n* = 499)	(*n* = 270)	(*n* = 229)	0.002
Uninsured	61 (12.2)	42 (15.6)	19 (8.3)	
Private	302 (60.5)	169 (62.6)	133 (53.2)	
Public	136 (27.5)	59 (21.9)	77 (33.6)	
Reported sexual partners	(*n* = 494)	(*n* = 281)	(*n* = 213)	
Cisgender men	185 (32.1)	99 (30.3)	86 (34.4)	0.293
Cisgender men only	46 (9.3)	28 (9.96)	18 (8.45)	0.566
Cisgender women	367 (63.6)	204 (62.4)	163 (65.2)	0.486
Transgender men	18 (3.1)	15 (4.6)	3 (1.2)	0.027
Transgender women	8 (1.4)	2 (0.6)	6 (2.4)	0.083
None	52 (10.7)	29 (8.9)	23 (9.2)	0.890
Gender‐affirming interventions	(*n* = 577)	(*n* = 327)	(*n* = 250)	
Hormones (testosterone)	455 (78.9)	243 (74.3)	212 (84.8)	0.002
Mastectomy	227 (39.3)	114 (34.9)	113 (45.2)	0.012
Metoidioplasty	6 (1.0)	3 (0.9)	3 (1.2)	0.525
Phalloplasty	4 (0.7)	1 (0.3)	3 (1.2)	0.321
Hysterectomy	53 (9.2)	27 (8.3)	26 (10.4)	0.230
Oophorectomy	45 (7.8)	21 (6.4)	24 (9.6)	0.158
Any of the above surgeries	240 (41.6)	121 (37.0)	119 (47.6)	0.010
HIV screening	250 (43.3)	–	–	
HIV positive	7 (1.21)	–	7 (2.8)	

Abbreviation: SD, standard deviation.

The majority of transmasculine individuals had used GAHT (455, 78.9%) and fewer had undergone surgical interventions (240, 41.6%). The most frequent GAS was mastectomy (227, 39.3%). Less than 2% had phalloplasty or metoidioplasty.

Transmasculine individuals had diverse sexual partnerships. The majority reported having at least one cisgender female sex partner (63.6%), while 32.1% reported at least one cisgender male partner. Forty‐six (9.3%) reported having cisgender male partners only.

### 3.2 HIV screening and prevalence

Less than half of transmasculine individuals in the sample had ever had an HIV screen (250, 43.3%). The Centers for Disease Control and Prevention and the United States Prevention Services Task Force recommend that clinicians screen patients at least once for HIV [19, 20]; therefore, HIV screening was suboptimal. This has been seen in previous research with transgender populations [21]; however, it was unexpected that this occurred in a health centre with a robust HIV programme and where all individuals presumably had good access to HIV and STI screening services.

Of screened individuals, 7/250 had a positive HIV test (2.8%; 95% CI 1.13%, 5.68%) (Table 1). HIV prevalence among screened individuals did not significantly differ by race/ethnicity, was likely due to the small sample size: Black individuals 3/44 (6.8%; 95% CI: 0.84%, 90.57%), Hispanic 1/31 (3.2%; 95% CI: 0.08%, 16.7%); other/multiracial 1/36 (2.8%; 95% CI: 0.07%, 14.53%) and White 2/96 (2.1%; 95% CI: 0.25%, 7.32%) (Table 1). HIV prevalence was highest for those with cisgender male partners only 2/18 (11.1%; 95% CI: 1.37%, 34.71%) (Table 2).

**Table 2:** HIV prevalence and gender identity of sexual partners^a^

Gender of sex partners	*n*	HIV positive	%	(95% CI)
Cisgender men only	18	2	11.1	(1.37, 34.71)
Cisgender men	86 (34.4)	3	3.49	(0.73, 9.86)
Cisgender women	163 (65.2)	3	1.84	(0.38, 5.28)
Transgender men	3 (1.2)	1	33.3	(0.84, 90.57)
Transgender women	6 (2.4)	0	–	–
No sexual partner	23 (9.2)	0	–	–
Declined to state	37	1	2.7	(0.07, 14.16)
Total screened for HIV	250	7	2.8	(1.13, 5.68)

^a^ Clients who were ever screened for HIV. Sexual partners listed are not mutually exclusive except where stated.

Abbreviation: CI, confidence interval.

In the bivariate analysis, living with HIV was associated with having a cisgender male partner only compared to those without cisgender male sexual partners (OR = 5.68, 95% CI 1.02, 31.58). Individuals with at least a high school diploma had reduced odds of HIV (OR = 0.07, 95% CI = 0.01, 0.49). Age, education and sex partner were placed into the multivariate model and both cisgender male partner only (OR = 10.58, 95% CI 1.33, 84.17) and high school diploma (OR 0.08, 95% CI 0.01, 0.72) remained significant predictors of HIV status (Table 3).

**Table 3:** HIV prevalence: bivariate and multivariable logistic regression models

	Bivariate		Multivariate	
Variables	OR (95% CI)	*p*‐value	OR (95% CI)	*p*‐value
Age in years	1.00 (0.92, 1.09)	0.977	1.04 (0.93, 1.15)	0.522
Race/Ethnicity				
White, non‐Hispanic	1.00	0.602	–	
Hispanic	1.63 (0.14, 18.65)		–	
Black, non‐Hispanic	3.56 (0.58, 22.26)		–	
Asian	–		–	
Other/multiracial	1.44 (0.127, 16.40)		–	
Employment status				
Employed	1.00	0.315	–	
Unemployed	2.19 (0.48, 10.06)		–	
Education				**0.009**
No high school diploma	**1.00**	0.007	**1.00**	
High school diploma	**0.07 (0.01, 0.49)**		**0.08 (0.01, 0.72)**	
Sexual partner(s)				**0.026**
No cisgender male sex partner	**1.00**	0.047	–	
Cisgender male sex partner only	**5.68 (1.02, 31.58)**		**10.58 (1.33, 84.17)**	
Gender‐affirming care				
No gender‐affirming care	1.00	0.583	–	
Gender‐affirming care	0.78 (0.09, 6.73)		–	
Gender‐affirming hormone therapy				
No hormones	1.00	0.946	–	
Hormones	1.08 (0.13, 9.21)		–	
Gender‐affirming surgery				
No gender‐affirming surgery	1.00	0.111	–	
Gender‐affirming surgery	0.18 (0.02, 1.49)		–	
Substance use				
No substance use	1.00	0.368	–	
Substance use	2.73 (0.31, 24.23)		–	

Note: Bolded ORs are statistically significant (*p* < 0.05).

Abbreviations: CI, confidence interval; OR, odds ratio.

The HIV prevalence found in this study is in line with a recent systematic review and meta‐analysis that estimated HIV prevalence among transgender men to be 1.2% by self‐report and 3.2% laboratory confirmed [13]. Although Becasen's review was unable to provide estimates by race or ethnicity for transgender men, data from the US National HIV Surveillance System reported that in 2019, 41% and 26% of newly diagnosed transgender men, and 45% and 22% of 461 transgender men living with HIV were Black and Hispanic, respectively [22].

These data revealed an HIV prevalence among transmasculine individuals who have sex with cisgender men that is substantially higher than the US population prevalence (0.41%) [23]. Previous research evaluating HIV prevalence among transgender men has not stratified results by the gender of sexual partners despite a wide range of reported sexual orientation identity [24] as well as sexual practices in this group [25], including higher rates of condomless sex, and numbers of sex partners among TMSM compared to those who do not [14, 15]. These findings have important implications for clinicians, researchers and policymakers, since transgender men are often not included in HIV prevention research and are not prioritized for HIV prevention intervention efforts [15, 26], which may contribute to their suboptimal utilization of HIV pre‐exposure prophylaxis (PrEP) [15, 27].

Our study had some important limitations. There have been important shifts over the last decade in the landscape of transgender health in the United States that limit the generalizability of these data. Public and commercial insurance coverage of gender‐affirming care has improved, in part, due to federal and state anti‐discrimination laws. There is greater visibility of transgender individuals, and those who identify as nonbinary, an identity not captured in these data. Other relevant changes include the routinization of HIV screening instead of risk‐based screening, as well as the implementation and scale‐up of HIV PrEP. The distribution of gender identities, prevalence of HIV screening and receipt of gender‐affirming care, therefore, reflect the period that these data were collected.

Additional limitations to this study include that clients were engaged in care at a single community health centre recognized for their transgender health programme and likely not representative of the US transgender male population.

EHR data on sexual practices beyond the genders of sexual partners were limited. Nonbinary identities for sexual partners were not recorded. These data did not capture sexual behaviours (i.e. receptive vaginal/receptive anal/receptive oral sex, etc.). Because of incomplete records, some data regarding HIV risk factors were missing, and it was also not possible to confirm whether data were current, for example for insurance data, housing status, and so on. It is possible that individuals may have been tested for HIV at other facilities and have not disclosed this fact to providers. The small number of individuals living with HIV, especially when categorized by race/ethnicity or gender of sexual partners, limited the ability to see significant/strong(er) associations.

## 4 CONCLUSIONS

This study of HIV prevalence among transmasculine individuals is the largest to date conducted at a community clinic in the United States. By stratifying HIV prevalence by the gender of sexual partners, this study adds important new information about HIV vulnerability among transmasculine individuals. These findings also underscore the need for improved inclusion of TMSM in HIV prevention research, which they have often been excluded from, and their recognition as a priority population for HIV prevention interventions.

## COMPETING INTERESTS

The authors declare no competing interests.

## AUTHORS’ CONTRIBUTIONS

AER performed the research. AER, ELL and MAC conceptualized the manuscript. AER analysed the data. AER wrote the first draft with assistance from ELL, MAC and ABH. All authors read and approved the final version.

## FUNDING

Research reported in this publication was supported by the National Institute of Mental Health under award number R25MH087217.

## Data Availability

The data that support the findings of this study are available from the corresponding author upon reasonable request.
